# 

**Published:** 1906-02

**Authors:** 


### THE AMERICAN
### Journal of Dental Science

**Founded, Owned, Edited and Published by the Dental Profession of America.**

**THIRD SERIES.**

**VOLUME XXXVII. FEBRUARY, 1906. NO. 2.**

EDITORS:

WM. GIRD BEECROFT, D. D. S., Madison, Wis. B. H. TEAGUE, D. D. S., Aiken, S. C.

Published on the 10th day of each month
Subscription Price, $1 00 per year. Foreign countries, $1.50 per year.

*Entered as Second-Class Matter, Madison, Wis.*

Address all communications, both business and editorial, to
**The Dental Publishing Company, Madison, Wisconsin.**

### SOCIETY ANNOUNCEMENTS.

Alabama State Dental Association meets in the Commercial Club rooms, Mobile, May 8th to the 10th. All ethical dentists urged to attend. One and one-third fare on all railroads on the certificate plan.

---

Florida State Dental Society meets at the Continental Hotel, Atlantic Beach (near Jacksonville), June 15th continuing three days. All ethical dentists urged to be present.

---

Kentucky State Dental Association meets at Dawson Springs, June 4th, 5th and 6th. A cordial invitation is extended to the profession to be present.

